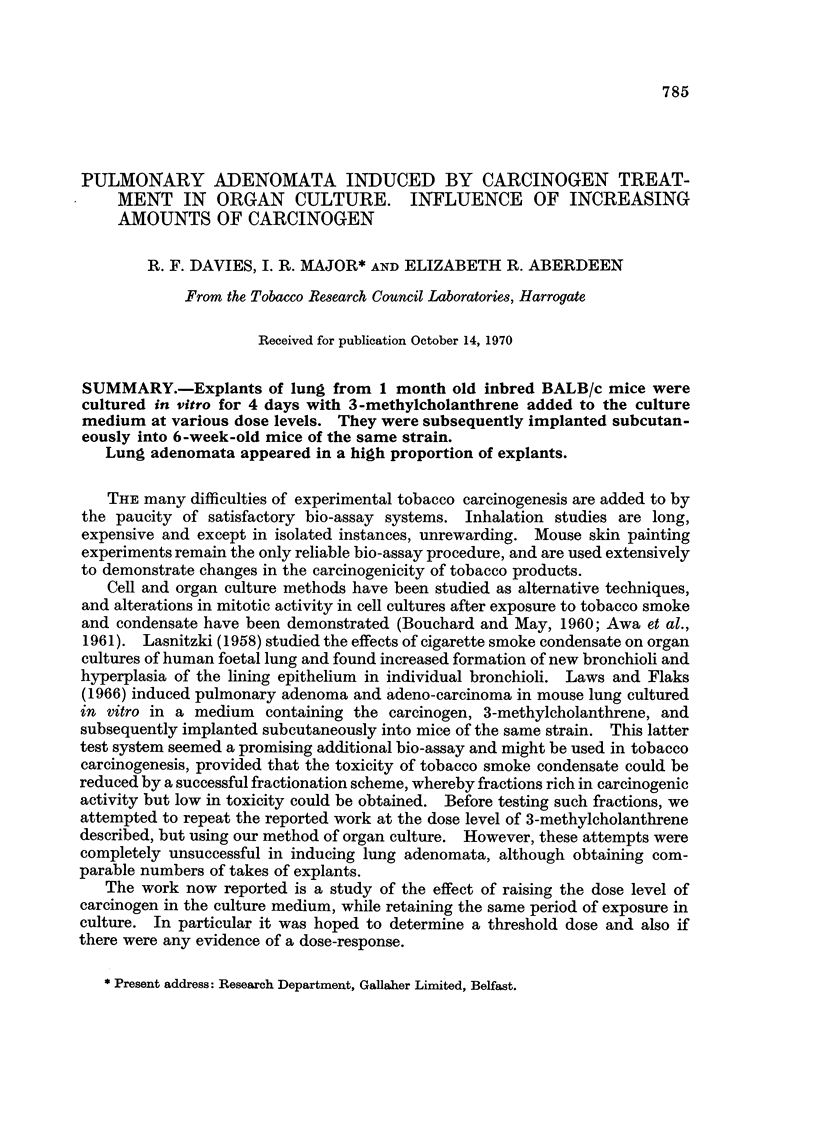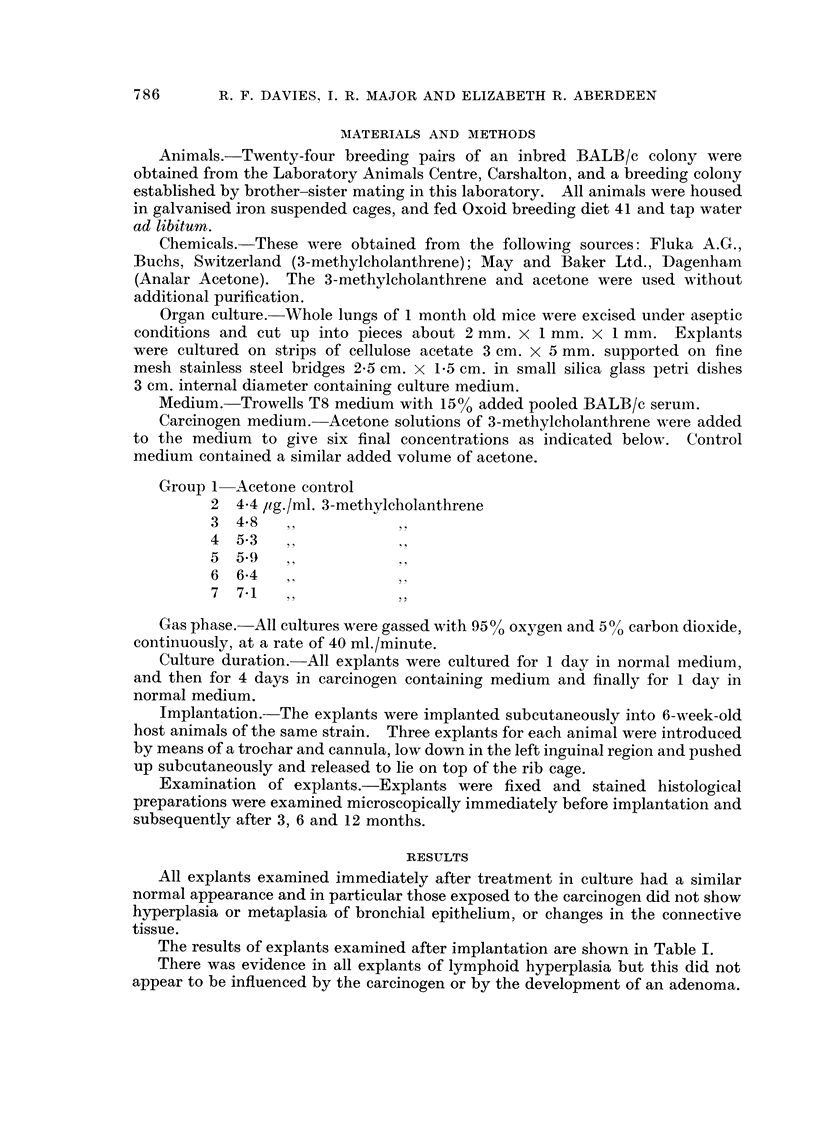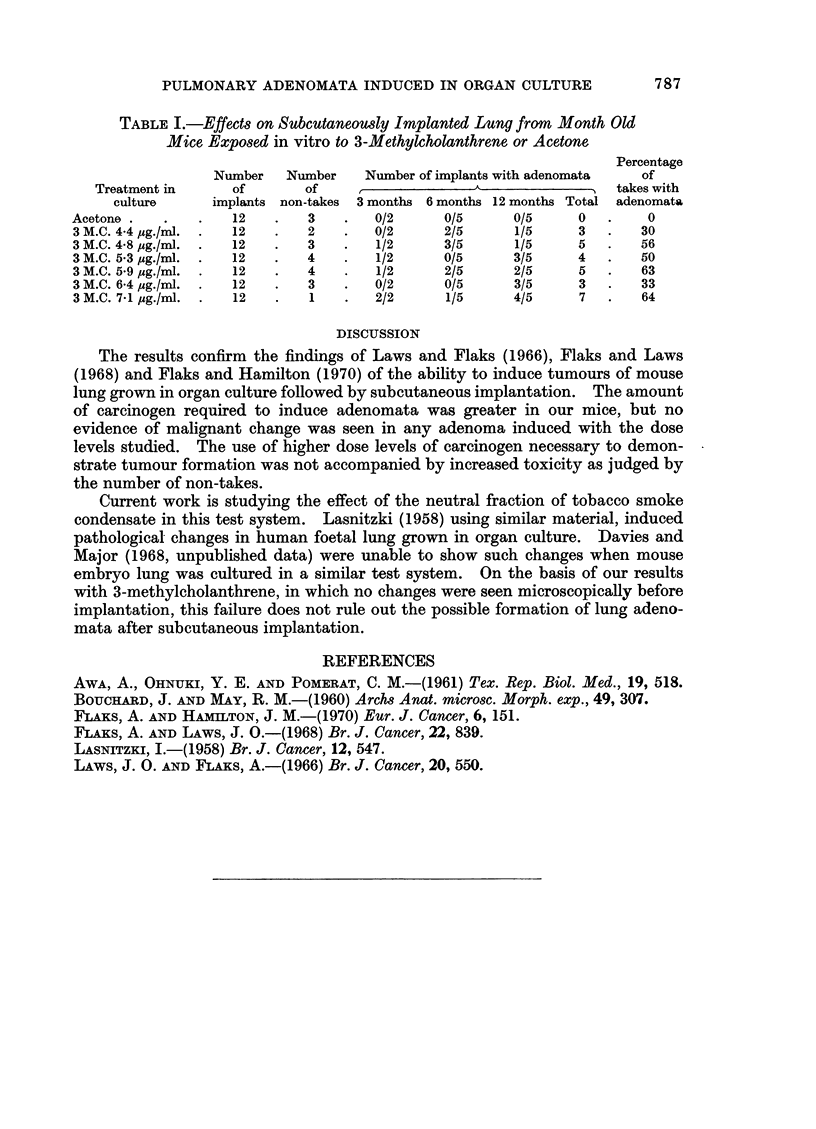# Pulmonary Adenomata Induced by Carcinogen Treatment in Organ Culture. Influence of Increasing Amounts of Carcinogen

**DOI:** 10.1038/bjc.1970.93

**Published:** 1970-12

**Authors:** R. F. Davies, I. R. Major, Elizabeth R. Aberdeen

## Abstract

Explants of lung from 1 month old inbred BALB/c mice were cultured *in vitro* for 4 days with 3-methylcholanthrene added to the culture medium at various dose levels. They were subsequently implanted subcutaneously into 6-week-old mice of the same strain.

Lung adenomata appeared in a high proportion of explants.


### PULMONARY ADENOMATA INDUCED BY CARCINOGEN TREATMENT IN ORGAN CULTURE. INFLUENCE OF INCREASING AMOUNTS OF CARCINOGEN

R. F. DAVIES, I. R. MAJOR* AND ELIZABETH R. ABERDEEN

*From the Tobacco Research Council Laboratories, Harrogate*

Received for publication October 14, 1970

**SUMMARY.—Explants of lung from 1 month old inbred BALB/c mice were cultured *in vitro* for 4 days with 3-methylcholanthrene added to the culture medium at various dose levels. They were subsequently implanted subcutaneously into 6-week-old mice of the same strain.**

**Lung adenomata appeared in a high proportion of explants.**

THE many difficulties of experimental tobacco carcinogenesis are added to by the paucity of satisfactory bio-assay systems. Inhalation studies are long, expensive and except in isolated instances, unrewarding. Mouse skin painting experiments remain the only reliable bio-assay procedure, and are used extensively to demonstrate changes in the carcinogenicity of tobacco products.

Cell and organ culture methods have been studied as alternative techniques, and alterations in mitotic activity in cell cultures after exposure to tobacco smoke and condensate have been demonstrated (Bouchard and May, 1960; Awa *et al.*, 1961). Lasnitzki (1958) studied the effects of cigarette smoke condensate on organ cultures of human foetal lung and found increased formation of new bronchioli and hyperplasia of the lining epithelium in individual bronchioli. Laws and Flaks (1966) induced pulmonary adenoma and adeno-carcinoma in mouse lung cultured *in vitro* in a medium containing the carcinogen, 3-methylcholanthrene, and subsequently implanted subcutaneously into mice of the same strain. This latter test system seemed a promising additional bio-assay and might be used in tobacco carcinogenesis, provided that the toxicity of tobacco smoke condensate could be reduced by a successful fractionation scheme, whereby fractions rich in carcinogenic activity but low in toxicity could be obtained. Before testing such fractions, we attempted to repeat the reported work at the dose level of 3-methylcholanthrene described, but using our method of organ culture. However, these attempts were completely unsuccessful in inducing lung adenomata, although obtaining comparable numbers of takes of explants.

The work now reported is a study of the effect of raising the dose level of carcinogen in the culture medium, while retaining the same period of exposure in culture. In particular it was hoped to determine a threshold dose and also if there were any evidence of a dose-response.

* Present address: Research Department, Gallaher Limited, Belfast.

### MATERIALS AND METHODS

Animals.—Twenty-four breeding pairs of an inbred BALB/c colony were obtained from the Laboratory Animals Centre, Carshalton, and a breeding colony established by brother–sister mating in this laboratory. All animals were housed in galvanised iron suspended cages, and fed Oxoid breeding diet 41 and tap water *ad libitum*.

Chemicals.—These were obtained from the following sources: Fluka A.G., Buchs, Switzerland (3-methylcholanthrene); May and Baker Ltd., Dagenham (Analar Acetone). The 3-methylcholanthrene and acetone were used without additional purification.

Organ culture.—Whole lungs of 1 month old mice were excised under aseptic conditions and cut up into pieces about 2 mm. × 1 mm. × 1 mm. Explants were cultured on strips of cellulose acetate 3 cm. × 5 mm. supported on fine mesh stainless steel bridges 2·5 cm. × 1·5 cm. in small silica glass petri dishes 3 cm. internal diameter containing culture medium.

Medium.—Trowells T8 medium with 15% added pooled BALB/c serum.

Carcinogen medium.—Acetone solutions of 3-methylcholanthrene were added to the medium to give six final concentrations as indicated below. Control medium contained a similar added volume of acetone.

| | | |
|---|---|---|
| Group 1 | —Acetone control | |
| 2 | 4·4 μg./ml. | 3-methylcholanthrene |
| 3 | 4·8 ,, | ,, |
| 4 | 5·3 ,, | ,, |
| 5 | 5·9 ,, | ,, |
| 6 | 6·4 ,, | ,, |
| 7 | 7·1 ,, | ,, |

Gas phase.—All cultures were gassed with 95% oxygen and 5% carbon dioxide, continuously, at a rate of 40 ml./minute.

Culture duration.—All explants were cultured for 1 day in normal medium, and then for 4 days in carcinogen containing medium and finally for 1 day in normal medium.

Implantation.—The explants were implanted subcutaneously into 6-week-old host animals of the same strain. Three explants for each animal were introduced by means of a trochar and cannula, low down in the left inguinal region and pushed up subcutaneously and released to lie on top of the rib cage.

Examination of explants.—Explants were fixed and stained histological preparations were examined microscopically immediately before implantation and subsequently after 3, 6 and 12 months.

### RESULTS

All explants examined immediately after treatment in culture had a similar normal appearance and in particular those exposed to the carcinogen did not show hyperplasia or metaplasia of bronchial epithelium, or changes in the connective tissue.

The results of explants examined after implantation are shown in Table I.

There was evidence in all explants of lymphoid hyperplasia but this did not appear to be influenced by the carcinogen or by the development of an adenoma.

TABLE I.—*Effects on Subcutaneously Implanted Lung from Month Old Mice Exposed* in vitro *to 3-Methylcholanthrene or Acetone*

| Treatment in culture | Number of implants | Number of non-takes | Number of implants with adenomata: 3 months | 6 months | 12 months | Total | Percentage of takes with adenomata |
|---|---|---|---|---|---|---|---|
| Acetone | 12 | 3 | 0/2 | 0/5 | 0/5 | 0 | 0 |
| 3 M.C. 4·4 μg./ml. | 12 | 2 | 0/2 | 2/5 | 1/5 | 3 | 30 |
| 3 M.C. 4·8 μg./ml. | 12 | 3 | 1/2 | 3/5 | 1/5 | 5 | 56 |
| 3 M.C. 5·3 μg./ml. | 12 | 4 | 1/2 | 0/5 | 3/5 | 4 | 50 |
| 3 M.C. 5·9 μg./ml. | 12 | 4 | 1/2 | 2/5 | 2/5 | 5 | 63 |
| 3 M.C. 6·4 μg./ml. | 12 | 3 | 0/2 | 0/5 | 3/5 | 3 | 33 |
| 3 M.C. 7·1 μg./ml. | 12 | 1 | 2/2 | 1/5 | 4/5 | 7 | 64 |

### DISCUSSION

The results confirm the findings of Laws and Flaks (1966), Flaks and Laws (1968) and Flaks and Hamilton (1970) of the ability to induce tumours of mouse lung grown in organ culture followed by subcutaneous implantation. The amount of carcinogen required to induce adenomata was greater in our mice, but no evidence of malignant change was seen in any adenoma induced with the dose levels studied. The use of higher dose levels of carcinogen necessary to demonstrate tumour formation was not accompanied by increased toxicity as judged by the number of non-takes.

Current work is studying the effect of the neutral fraction of tobacco smoke condensate in this test system. Lasnitzki (1958) using similar material, induced pathological changes in human foetal lung grown in organ culture. Davies and Major (1968, unpublished data) were unable to show such changes when mouse embryo lung was cultured in a similar test system. On the basis of our results with 3-methylcholanthrene, in which no changes were seen microscopically before implantation, this failure does not rule out the possible formation of lung adenomata after subcutaneous implantation.

### REFERENCES

AWA, A., OHNUKI, Y. E. AND POMERAT, C. M.—(1961) *Tex. Rep. Biol. Med.*, **19**, 518.
BOUCHARD, J. AND MAY, R. M.—(1960) *Archs Anat. microsc. Morph. exp.*, **49**, 307.
FLAKS, A. AND HAMILTON, J. M.—(1970) *Eur. J. Cancer*, **6**, 151.
FLAKS, A. AND LAWS, J. O.—(1968) *Br. J. Cancer*, **22**, 839.
LASNITZKI, I.—(1958) *Br. J. Cancer*, **12**, 547.
LAWS, J. O. AND FLAKS, A.—(1966) *Br. J. Cancer*, **20**, 550.